# Perception and Experience of Transcultural Care of Stakeholders and Health Service Users with a Migrant Background: A Qualitative Study

**DOI:** 10.3390/ijerph181910503

**Published:** 2021-10-07

**Authors:** Benjamin Gaya-Sancho, Valérie Vanceulebroeck, Nuran Kömürcü, Indrani Kalkan, Antonio Casa-Nova, Elena Tambo-Lizalde, Margarida Coelho, Evy Present, Seda Değirmenci Öz, Teresa Coelho, Sofie Vermeiren, Arzu Kavala, Benjamin Adam Jerue, Berta Sáez-Gutiérrez, Isabel Antón-Solanas

**Affiliations:** 1 Department of Nursing, Faculty of Health Sciences, Universidad San Jorge, 50830 Zaragoza, Spain; bajerue@usj.es (B.A.J.); mbsaez@usj.es (B.S.-G.); 2 Research Group of Research on New Targets in Autoimmunity and Oncological Surveillance (INDIVO) (B3_20D), Universidad San Jorge, 50830 Zaragoza, Spain; 3 Department of Nursing, AP University of Applied Sciences and Arts, 2000 Antwerpen, Belgium; valerie.vanceulebroeck@ap.be (V.V.); evy.present@ap.be (E.P.); sofie.vermeiren@ap.be (S.V.); 4 Faculty of Health Sciences, Istanbul Aydin University, Istanbul 34295, Turkey; nurankomurcu@aydin.edu.tr (N.K.); indranikalkan@aydin.edu.tr (I.K.); sedadegirmenci@aydin.edu.tr (S.D.Ö.); arzukavala@aydin.edu.tr (A.K.); 5 Instituto Politécnico de Portalegre, School of Health Sciences, 7300-110 Portalegre, Portugal; casanova@ipportalegre.pt; 6 Instituto de Investigación Sanitaria, Hospital Universitario Miguel Servet, 50009 Zaragoza, Spain; etambo@salud.aragon.es; 7 Instituto Politécnico de Portalegre, School of Education and Social Science, 7300-110 Portalegre, Portugal; margco@ipportalegre.pt (M.C.); teresa.coelho@ipportalegre.pt (T.C.); 8 Department of Physiatry and Nursing, Faculty of Health Sciences, Universidad de Zaragoza, 50009 Zaragoza, Spain; ianton@unizar.es; 9 Research Group Nursing Research in Primary Care in Aragón (GENIAPA) (GIIS094), Institute of Research of Aragón, 50009 Zaragoza, Spain

**Keywords:** nurses, cultural competency, healthcare system, qualitative research, transcultural nursing, culture awareness

## Abstract

Introduction: While European health policies do frequently take into consideration the ideas and experiences of their users, the voices of minority and marginalized communities are not often heard. European healthcare services must address this issue as the number of healthcare users with an MM background increases. Aim: To explore the perspectives of key stakeholders and healthcare users with an MM background on transcultural care in four European countries. Design: Qualitative phenomenological study. Methods: Semi-structured, individual interviews were conducted with stakeholders and MM users. Interviews were translated and transcribed verbatim and were carried out from February to May 2021. Descriptive statistics was used to describe the characteristics of the sample; qualitative data were analyzed thematically following Braun and Clarke’s phases, resulting in 6 themes and 18 subthemes. Results: For stakeholders and MM users with long-established residence in their respective countries, cultural differences involve different family and community norms, religious beliefs, lifestyles, and habits. These components are perceived as in tension with healthcare norms and values, and they mediate in two key and related aspects of the relationship between MM users and healthcare providers: accessibility and communication. Conclusions: Communication and access to healthcare are key to MM health service users, and they are the most frequent sources of misunderstanding and conflict between them and healthcare professionals. Impact: It is important to extend the investigation of cultural issues in healthcare to stakeholders and MM users. There is no doubt that healthcare professionals should be trained in cultural competence; however, cultural competence training is not the only area for improvement. There should be a change in paradigm in healthcare services across Europe: from individual to organizational integration of culture and diversity.

## 1. Introduction

This paper was developed as part of a European project, which arose from the need to stop social exclusion and inequality emerging in Europe considering the European Union’s (EU) social policy aims “to promote employment, improve living and working conditions, provide an appropriate level of social protection and develop measures to combat exclusion” [1]. The project was funded by the Erasmus+ program under Key Action 203 Strategic Partnership for Higher Education, and its aims are to foster the development of social, civic, and transcultural competences and to tackle discrimination, segregation, racism, bullying, and violence in healthcare by investigating the current situation and proposing a new approach for nurses. It represents a collaboration between four European universities. In this paper, we present the findings of a qualitative study to analyze the perspectives of key stakeholders and decision makers, as well as those of healthcare users from a diverse cultural background, on transcultural care in four European countries to facilitate the relevant points that will allow a better approach to the emerging problem. In addition, the originality of the article lies in the comparison between the groups studied together with the content of the paper.

### 1.1. Background

Issues surrounding migrant health and healthcare have been central and controversial issues in European policy circles for years [2], and this continues to be the case seeing that the number of people with a migrant background living in Europe continues to increase [3]. Over the last few decades, some countries of the European Union have all experienced meaningful demographic changes [4]. Once characterized by their homogeneous society, they have now become multicultural countries striving to adapt to the new state of affairs. The reasons behind this transformation are diverse and vary slightly from one nation to another. For instance, Spain, Turkey, and Portugal are geographically in close proximity to the African continent, one of the main sources of immigration to the EU, yet there are other more complex contributing factors for the recent rise in the number of migrants and refugees, including the steady rise of most EU countries’ national gross domestic product (at least until recently), Spain, Portugal, and Belgium’s colonial histories, and Turkey’s shared border with Syria.

While immigration can be seen as culturally enriching to the host society, it can also lead to overcrowding, religious and cultural conflict, and increased education and health costs. These and other factors may contribute to widening the gap between the majority population and those from a migrant minority (MM) background, resulting in worse access to healthcare, housing, education, and work. In fact, results from previous research have shown that health disparities between minority and non-minority groups do exist. Cultural and social determinants to health, such as unequal access to care, poverty, racism, failings of intercultural communication, and ineffective interactions between the care provider and the patient, have been identified as some of the factors underlying these [4,5,6,7,8,9,10,11,12].

In the past few decades, initiatives have been taken to address these issues. Recently, in 2018, the European Commission’s Expert Panel on effective ways of investing in health (EXPH) proposed several actions to ensure equity in access to healthcare: (1) People should have access to proper health information and services information in their own language; (2) health services should meet the needs of all by being culturally sensitive and appropriate; (3) healthcare services should provide interpretation and translation facilities, teaching diversity in the health workforces and adaptation of diagnosis and treatment methods, to be culturally appropriate and acceptable, all of which are key aspects of cultural competency [13]. However, despite the identification of real problems and proposed best practices, the implementation of such care interventions remains patchy [14]. According to Teunissen et al. [14], this can be explained by the fact that health services are not tailored to day-to-day practice, and users from minority groups are not being involved in the decision-making process. However, this knowledge–practice gap is under-researched and should be investigated further [15,16,17].

While it is common for national health policies to take into consideration the ideas and experiences of their users, the voices of minority and marginalized communities are not often heard [18]. People from an MM background are frequently considered “hard to reach” [17] due to lack of accessibility, language difficulties and cultural difference, and cultural mismatches, which are precisely some of the specific challenges that these users face when accessing healthcare cultural competence in healthcare [19]. These must be considered as the barriers to cultural competence in healthcare [19].

Social and cultural determinants of health identify the key aspects that influence the health status of people belonging to cultural minorities [20]. European healthcare services must address this issue as the number of healthcare users with an MM background increases [21], considering that Global Health Europe states certain European health values such as a scientific mindset, equity, and universality, in which scholars still detect gaps and where there is still a delay in the adaptation of health services to diversity and equity [22] not related to cultural competence but to the desire for cultural matching [23,24].

### 1.2. Aim

As part of this broader framework, this qualitative study aims to explore the perspectives of key stakeholders, decision makers, and healthcare users with an MM background on transcultural care in order to understand their views on the design, integration, and implementation of strategies to provide a culturally mindful and safe healthcare [25] for all in some European countries.

## 2. Material and Methods

### 2.1. Design

As the research aims to elicit participants’ perception of transcultural care, a qualitative phenomenological approach was selected. This is appropriate as phenomenological research is a qualitative research method which seeks to understand complex phenomena through the participants’ lived experience, meaning, and perspectives [26,27].

The COREQ reporting guidelines [27] were used in both the framing and reporting of this study to guarantee that sufficient detail on the methods of data collection, analysis, and interpretation is provided (Table S1).

### 2.2. Participants

The study target population consisted of two main groups, namely, key stakeholders and decision makers in healthcare and individuals with an MM background from Spain, Belgium, Turkey, and Portugal. The participants in each group were selected following a purposive sampling technique. Access to the sample was gained through previously identified gatekeepers, namely, coordinators and directors from healthcare organizations and services, healthcare service user groups, and local NGOs. We ensured that the researchers who conducted the interviews were not personally acquainted with the interviewees. Although the interviews were not entirely free of the subjectivity of both the interviewer and the interviewee, every attempt was made on our part to ensure that the participants felt free to discuss their personal views and opinions on the study phenomenon.

Specifically, 14 key stakeholders and decision makers and 19 participants with an MM background who were representative of a minority group of each country’s population were selected according to the inclusion criteria (Table 1).

We excluded from our sample any participants who could not communicate in the official language of each country or in English, and those who refused to give informed consent.

### 2.3. Methods of Data Collection

The process of data collection took place simultaneously in all four study sites from February to May 2021.

The participants were invited to complete a sociodemographic questionnaire with the aim of describing the characteristics of our sample, including the following variables: age (years), gender, race/ethnicity, country of birth, country of work, and level of education. In the case of the participants with an MM background, the following variables were added to the list: marital status, religious affiliation, socioeconomic level, country of residence, and years living in that country.

One-to-one interviews with the participants took place either online or in person depending on the participant’s preference and the COVID-19 pandemic situation in each country. The interviews lasted around 60 min approximately. This method of data collection was chosen because it can elicit rich, culturally grounded insights into people’s perspectives and experiences [28]. All of the data were audio or video recorded and transcribed verbatim. The results were subsequently translated into English by the academic conducting the interview, except for the Spanish interviews that were translated by the researcher who carried out the data analysis and interpretation, all of whom are fluent in English. All the researchers used the same (previously agreed) interview guides developed by the principal investigator (IA-S). Some of the items in this interview guide were adapted from the “Evaluation Guide: Practical Strategies for Culturally Competent Evaluation” [29]. Interview questions addressed topics such as the involvement of groups from an MM background in decision making in healthcare and the strengths and challenges in the implementation of culturally safe health services (see Appendix A and Appendix B). The interviewers followed the interview guides and were able to modify and include those aspects that they considered relevant according to the content discussed during the interview.

### 2.4. Data Analysis

Descriptive statistics was used to analyze the sociodemographic data using frequency and percentage for qualitative variables and mean and standard deviation for quantitative ones.

The anonymized transcripts were analyzed qualitatively following Braun and Clark’s [30] phases for thematic analysis, namely, familiarizing with data, generating initial codes, searching for themes, reviewing themes, defining and naming themes, and producing the report.

Six themes and eighteen subthemes emerged from thematic analysis of the transcripts (Table 2). A summary of the themes and subthemes identified, illustrated by representative examples of quotes, is presented in Appendix C (Stakeholders) and Appendix D (MM users). The order in which the topics are presented is not correlative but is presented to guide the reader through the results. From topic 5, subtheme 5.2, onwards, the separation between Stakeholders and MM users is not defined because the concepts were the same for both groups.

### 2.5. Validity and Reliability

Two researchers analyzed the transcripts and identified themes and subthemes from the data, guaranteeing trustworthiness and enough information power (saturation) with the available interviews.

### 2.6. Ethical Considerations

Approval from the Research Ethics Committee of the Community of Aragon (CEICA) was granted on 31 January 2021 (C.P.-C.I. PI20/607). All the participants agreed to take part in the study following an explanation of its aims and procedures and signed the consent form before being interviewed. Participant anonymity and confidentiality were guaranteed and safeguarded, complying with General Data Protection Regulation (RGPD 2016/679).

## 3. Results

The sociodemographic and cultural characteristics of our sample are presented in Table 3.

The sample of stakeholders and decision makers comprised 9 women and 5 men, mostly aged 40–50. Over 70% of the participants had higher education level studies; three were educated to vocational training level and one to secondary education level. All the participants but one defined themselves as being White, and all of them were working in the same country where they had been born.

A total of 19 healthcare service users with an MM background, 12 women and 6 men, agreed to be interviewed. Most of the participants in this group were aged 20–30, and about half of them defined themselves as being either low or middle class. Our participants’ ethnic and religious backgrounds were mixed, including people from a White (26.3%), mixed (15.7%), Asian (15.8%), and Black (31.6%) ethnic background; individuals practiced Islam (42.1%), Catholicism (36.8%), Judaism (10.5%), or they did not practice any religion (10.5%). The MM users’ places of origin were varied except for the case of Turkey, where all the interviewees came from Bangladesh (21.05%). Regarding the time living in their respective countries of residence, most of the interviewees had been living in Spain (21.05%), Portugal (21.05%), Belgium (36.84%), and Turkey 21.05%) between 16 months and 6 years before being interviewed. One of the participants living in Spain and all the participants living in Belgium said that they had been living in their current country of residence for a long time or all their lives. The MM users’ level of education was slightly lower than that of the stakeholders, with a third being educated to secondary school level (31.58%), a third to higher education level (31.58%), and just over 20% to vocational training level (21.05%).

The results from the qualitative analysis are presented below. For the cases where the perceptions and experiences of the stakeholders were different from those of the MM users, the results are presented separately; for the cases where their perceptions and experiences were similar, the results are presented jointly.

### 3.1. Theme: Concept of Culture

It is important, before analyzing the participants’ experience of cultural diversity in healthcare, to describe their understanding and concept of culture.

#### 3.1.1. Subtheme: Meaning of Culture

*Stakeholders*: Culture is associated with origin, ethnicity and nationality, race, beliefs and behaviors, and sense of belonging to a community. Culture is also viewed both as conflictual and as a value in itself.

“Life trajectory, place of origin, place where people have grown up (determines) beliefs and values” (S8)

*MM users*: A clear definition of (one’s) culture is more evasive in people with MM backgrounds’ accounts. It has to do with habits, external signs, and language, and it is in tension with equal treatment.

“I wear the veil, but they do not look at me as if I was alien, I do not know” (M11)

#### 3.1.2. Subtheme: Impact of Culture on Health

*Stakeholders*: The majority of stakeholders identified culture with habits and lifestyles that can negatively influence health. They also view culture as determining conceptions of illness that lead to healthcare problems, such as lack of adherence to treatment.

“I think that people as they have different ways of thinking have different beliefs, different life habits and different types of eating. All these aspects have an impact on people’s lives and can affect these minorities negatively” (S12)

*MM users*: In consonance with the evasive approach to culture in subtheme 1.1, most of interviewees did not directly associate culture with health. However, although not identified explicitly as culture-related, some of their responses did make reference to aspects such as biological determinants of health, communication issues and their impact on health, and internalized notions of healthcare as a right.

“For the migrant people that are here (Spain) for less than a year, there are a lot of difficulties (…) First, to regularize their situation, second, to think of themselves as having rights. There could be a part that has to do with the place of origin, where maybe the conception of who is a rights’ bearer is not internalized as much” (M8)

### 3.2. Theme: Impact of Culture on Healthcare Experience

Our participants reflected on those factors shaping their current experience of designing and/or managing, or using, healthcare services and described the cultural components included within such factors. Specifically, while the stakeholders gave importance to specific cultural aspects including habit and tradition, religion, beliefs, gender norms, and language, among others, MM users did not make an explicit reference to these or other characteristics. However, the participants in both groups described culture as a source of misunderstanding and conflict. Interestingly, the stakeholders were generally more “straightforward” when describing their ideas and opinions on this matter, whereas the MM users adopted a more nuanced discourse.

#### 3.2.1. Subtheme: Perception of Own Culture and Its Impact on Healthcare Experience

*Stakeholders*: The participants did not necessarily describe their own culture, but they stressed values and institutional norms in healthcare as opposed to cultural norms of minorities—for example, openness, secularism, science and medicine, and universal access to healthcare.

“We use the Biomedical Model a lot in the process of decision making. It is difficult to people from diverse cultural backgrounds to participate in their own decision making” (S10)

*MM users*: The participants did not stress their own culture as playing a key role in their healthcare experience, but some aspects of belonging to a cultural minority, namely, language, religion, and problems to access healthcare due to migratory status, were perceived by some as a barrier to healthcare.

“It is difficult (for healthcare staff) to be aware of small customs of other cultures (…) I do not think they should be prepared for such small things (…) Perhaps provide activities for patients who receive few visitors, definitely during the corona pandemic. The food could perhaps also be improved” (M4)

#### 3.2.2. Subtheme: Perception of the Other’s Culture and Its Impact on the Healthcare Experience

*Stakeholders*: Certain culturally influenced attitudes and behaviors were perceived as in tension with healthcare service patterns and guidelines. Culture of minorities was perceived as communitarian and family based, which stakeholders saw as clashing with institutionalized care and continuity of care.

“I know that they restore to healthcare in acute pathology situations and then, follow-up in terms of primary healthcare does not happen (…) we lose track to these minority groups” (S10)

*MM users*: Living in a multicultural community was identified as a positive determinant of MM users’ experience of healthcare, whereas frequently repeated “cultural mantras”, such as the notion that “migrants abuse the health service”, were described as having a negative impact.

“There is a mantra that the immigrants use healthcare more than anyone else (…) and that generates that immigrants do not think of themselves as worthy of such assistance” (M8)

#### 3.2.3. Subtheme: Culture as a Source of Misunderstanding and Conflict

*Stakeholders*: The majority identified conflicts stemming from a climate of fear and mistrust between healthcare professionals and MM service users. In addition, the stakeholders referred to certain attitudes and behaviors displayed by patients from culturally diverse backgrounds that, in their view, clashed with what was perceived by them as being basic healthcare principles and even good professional practice.

“It has been difficult for me (…) because you reach a level of vehemence on both parts, the lack of understanding, it is difficult for me to understand, for example, when there is someone sick and a lot of family members from gypsy race come, they are culturally like that, but this clashes with norms and things that are not allowed for other people” (S5)

*MM users*: The participants did not stress conflict, but they mentioned some factors that might lead to it, namely, language, communication difficulties, personal attitudes, and different expectations about healthcare.

“To listen and be listened (to). If you are going to talk to a doctor, and he or she is in another world, the patient gets angry, because you see in the doctor’s face that he/she is not with you” (M11)

### 3.3. Theme: Discrimination and Racism

Most of our participants identified instances of discrimination in relation to key healthcare principles including access, information, and equal treatment. However, they insisted that they had not personally been discriminated against nor witnessed any examples of discriminations against others, and that the treatment received from healthcare professionals was not discriminatory. Examples of racist comments and/or treatment were recalled by the participants in both groups, but the MM users avoided labeling them as racist.

#### 3.3.1. Subtheme: No Personal Experience

*Stakeholders*: None of the participants identified situations in which they exercised or witnessed discrimination.

“(We treat everyone), even the undocumented, and there is no problem” (S7)

*MM users*: None of the participants said that they had been discriminated against while using the healthcare services, but they did admit that they had experienced discrimination in other contexts. Additionally, most of them had heard stories about other MM users experiencing discrimination in the healthcare context.

“(I faced) discrimination as an intern, but as patient no (…) Everyone is equal, and everyone gets the same access to health services.” (M6)

#### 3.3.2. Subtheme: Discrimination and Equal Access/Treatment

*Stakeholders*: Some of the participants in the group of stakeholders described examples of MM users receiving worse quality information, having unequal access to healthcare, and being prejudiced against.

“When a Gipsy family comes (…) there is suspicion, a predisposition to conflict (…) “let’s see how they behave” (S5)

*MM users*: The majority of healthcare users from an MM background identified problems accessing healthcare and said that they were mistreated by administrative staff.

“(With language) some people (staff) don’t have much patient in that (…) (A foreign name can cause to offer help) less enthusiastically” (M2)

#### 3.3.3. Subtheme: Racist Treatment

*Stakeholders*: The participants recalled racist comments from professionals and from other patients toward people from cultural minorities.

“The biggest challenge has to do with a smaller tolerance on the part of some people in Portuguese society, (…) Many Portuguese citizens have the perception that these people who come from outside, have more rights over their own nationals and, this is not quite clear, the Portuguese feel that they themselves are being discriminated against other people from minorities” (S9)

*MM users*: The interviewees avoided describing specific attitudes and behaviors from healthcare staff as being racist, and instead, they recalled examples of racism between patients.

“I do not know if this racism or not, but because she was a Muslim, a girl’s wearing the veil (…) that person (the doctor) wants to end it, does not respond to questions, maltreat, and such” (M9)

### 3.4. Theme: Integrating Social Diversity in Care

Our participants reflected on ways of integrating cultural minorities in the design, implementation, and delivery of care. All agreed on the fact that, currently, there is no involvement, and if there is, it is at the user level. There were doubts among some participants about whether such effort of integration was needed, or if there were things that needed to be fixed first, namely, waiting lists and universal access to healthcare. While healthcare users from an MM background were generally happy with the professionals’ training, the stakeholders insisted on the need for more cultural education and training, for an enhanced level of cultural awareness, and for a change in attitude.

#### 3.4.1. Subtheme: Importance of Diversity in Healthcare

In the interviews, participants were asked about the importance of integrating cultural diversity in healthcare. In their responses, they reflected on what that process meant to them and what they believed were the main factors fostering and obstructing such integration.

*Stakeholders*: Generally, the stakeholders understood that there was a balance to be achieved between giving the same care to everyone and adapting care to people’s needs for cultural reasons. Some suggested implementing a more individualized and proactive approach to care as a way to improve healthcare in general and for all. Two Spanish participants explicitly mentioned the importance of equity within the healthcare system, especially primary care.

“Care should be generic, there should be an equal offer for everyone. Personal values and norms among different cultures must be taken into account” (S3)

*MM users*: Doubts on the importance of integrating cultural diversity in healthcare were expressed, and participants identified other priorities, such as the need for more empathy, time, active listening, and universal access to healthcare.

“One small point of work is that the caregivers are not always empathetic. Of course, it is very busy now, so I understand that there is often little time, but there is still room for improvement in terms of individual treatment so that the patient feels understood and worthy. For example, they should not be afraid to ask questions. Other than that, I have no major minuses, I only have pluses (laughs)” (M6)

#### 3.4.2. Subtheme: Education and Training of Healthcare Professionals

*Stakeholders*: While the participants stressed the importance of ensuring that healthcare professionals received systematic training in cultural diversity, they also insisted on the importance of both attitude and previous personal experiences; some even made the first dependent on the second to produce results.

“There is not so much training as quality of the person” (S8)

*MM users*: The participants in this group had nuanced views on this topic, expressing satisfaction with the healthcare professionals’ level of training, as well as identifying some areas of improvement, such as language and knowledge of, and sensibility toward, other cultures.

“There is no course on culture, or transcultural (…) so it is the sensibility you have as an individual, if you wanted to get a training yourself, or your personal experience (…) I think there should be internal training for professionals” (M8)

### 3.5. Theme 5: Barriers Accessing Healthcare, and Limiting Quality of Care

Both the stakeholders and the service users from MM backgrounds agreed on the current barriers affecting patients from culturally diverse backgrounds’ access to healthcare and limiting the quality of care received once access was granted. Specifically, language difficulties were mentioned frequently by the participants in both groups. In addition, some reflected on other communication barriers and sources of misunderstanding stemming not only from the meaning of words but from differences between the healthcare professionals and the patients’ worldviews. Institutional barriers such as exclusion from regular healthcare for administrative reasons, lack of time, lack of diversity within the healthcare leadership and workforce, lack of information, and lack of socioeconomic resources were also identified.

#### 3.5.1. Subtheme: Communication and Language

All participants mentioned language as one of the main problems when asked about integrating MM users in healthcare. However, the majority of them also went further and reflected on communication, which includes language but also understanding each other’s meanings.

*Stakeholders*: the majority of participants saw language and communication as key to trust-based interactions in healthcare.

“Mutually, there are problems based on trust (…) there are difficulties in providing services due to communication and trust problems” (S14)

*MM users*: for our participants, difficulties communicating had to do with language, but also with healthcare terminology, and with professionals’ disposition to listen to them.

“I have faced the difficulties in communicating with the healthcare professionals. Here, Turkish language is the official language and professionals didn’t have enough skills in English especially in the public hospitals” (M16)

#### 3.5.2. Subtheme: Institutional Barriers

Both groups of participants reflected on the exclusion of minorities from primary care as one of the main barriers to integrating social diversity in healthcare. Some stakeholders mentioned their lack of time to dedicate to patients, especially when there are problems of communication, while an MM user reflected on the lack of knowledge that healthcare institutions and professionals have of migrants’ experiences.

“In the care sector, time is the problem. If you don’t make time for people who are not used to care in Belgium, combined with the language barrier, this can cause problems” (S2)

“(…)When you come here (Spain), this happens a lot, every person with a uniform, be it police be it a healthcare uniform, exercises a power, a reaction (on the part of the migrant) of submission and fear (…) there are civil servants in one rail, and in another the migrant population, and there are clashes” (M8)

#### 3.5.3. Subtheme: Diversity within Staff

Stakeholders and MM users mentioned the small presence of people from different cultural backgrounds and migrant backgrounds in healthcare. As one MM user reflected, this can impact quality of care.

“You notice that also in other institutions such as education, there are few people with a different background working there” (S4)

“Often my language is not good enough to communicate with Belgian healthcare providers, so sometimes I go to Polish healthcare providers, but because there are only a few Polish healthcare providers, they are often very busy, so the quality of care is not so good. That’s the reason why we changed GPs” (M6)

#### 3.5.4. Subtheme: Resources Accessing Healthcare

Participants identified resources that were lacking and that were important to integrate social diversity in healthcare: time to dedicate to patients, more professionals to reduce waiting lists, and lack of information and education on how the system functions. One stakeholder mentioned lack of economic resources on the part of MM users as a barrier to a positive healthcare experience, and one MM user mentioned lack of personal contact with her physician due to COVID-19 restrictions as a barrier.

“If they don’t get the education around it (health and healthcare) as much, you notice bigger problems there” (S1)

“I had a bladder infection last year (…) I had explained my situation on the phone to the Polish GP and went for a consultation afterwards. I missed the contact and interaction with the doctor, which made me feel like one of many patients” (M6)

### 3.6. Theme: Facilitators

In line with what was discussed about barriers, stakeholders and MM users generally agreed when identifying facilitators to integrate cultural factors in healthcare. Our participants reflected on how people are already using what they have to bridge the gap in communication (internet, phone apps, religious and cultural leaders, and family members and friends as informal translators). They also supported hiring more translators and cultural mediators in healthcare settings. The participants also mentioned examples of institutional orientation toward the inclusion of cultural minorities, both as patients (at least at the user level) and as healthcare practitioners. Finally, one participant insisted in the need for training patients, so they can exercise their right to healthcare.

#### 3.6.1. Subtheme: Communication and Language

Participants reflecting on communication and language proposed similar solutions to such problems: increase resources to improve not only translation, but also the communicational aspect of the patient–professional relationship. Hiring translators but also cultural mediators were the most common proposals.

“Of course, we can’t have a translator in every hospital in every health center but maybe we should bet a little more on these translation support telephone lines in different languages in a 24-h…” (S10)

“There are more people from migrant origin that are doctors, nurses, there is a change that is going to happen (…) But we are talking about a very long process that is very slow. What can be done in the meantime? Think about what we are doing and changing it, for example, with translators (…) cultural mediators, social educators” (M8)

#### 3.6.2. Subtheme: Institutional Orientation

Participants reflected on how healthcare as an institution could orient itself toward better integrating social diversity. Stakeholders addressing this issue mentioned the importance of empathy and of people’s disposition to learn from each other as key components of such orientation. Some stakeholders and MM users also mentioned individualized care. One MM user insisted on an institutional orientation of healthcare as a right for all.

“Yes, a growth in empathy. You have to be able to imagine very well why someone wants or doesn’t want certain things (…) the care does not immediately become better, but existentially it does” (S3)

“There is a need for more individual support (…) individualized care” (M2)

#### 3.6.3. Subtheme: Diversity within Staff

As mentioned above, there was an agreement between stakeholders and MM users in thinking that hiring more professionals with an MM background would be a facilitator to integrate social diversity in healthcare.

“Diversity within social workers. More people from cultural backgrounds need to be hired. Patients look up to them and have confidence in them. Language skills are also a challenge” (S3)

“I actually think it’s positive when many different cultures work together. The diversity ensures that people are helped better. People from different origins working as nurses or bakers, for example” (M2)

#### 3.6.4. Subtheme: Resources That Facilitates

Participants identified institutional resources that could improve the integration of social diversity in healthcare. Although with different names, the majority of stakeholders mentioned entry points, or points of contact, to reach MM users and follow them, and one advocated for a de-digitalization process to improve accessibility. MM users reflected on the resources they were already using to facilitate their access to, and experiences with, healthcare, signaling possibilities for institutional resources.

“It is important to have a point of contact for people from different cultural backgrounds. This point of contact can serve as a navigation that can help the patient further. For the vulnerable groups there should be proactive care, people who are actively involved in the care of certain patients and who follow them up systematically” (S3)

“People reach to their cell phones very quickly. People who are older, like my parents, rely on their children to help them (…) the Internet is also used, often the Facebook group is used, and people help each other (with documents) (…) Real resources are not used much, but we mainly help each other” (M6)

## 4. Discussion

In this paper, we analyzed the perception of culture held by stakeholders and healthcare users with an MM background, as well as their experiences with healthcare in general, and with cultural diversity in healthcare in particular.

We asked our participants to define culture and its relationship with health in their own words. Some of the stakeholders clearly related the concept of culture to particular aspects attributed to cultural minorities such as race, ethnicity, and place of origin, a view of culture shared by Reneau [31]. However, in agreement with other authors’ views [26,32], most of the stakeholders extended this notion of culture to introduce elements such as religion, beliefs, gender norms, age and generation, and habits and lifestyles. Others such as Phillmore [22] prefer to use the notion of diversity to include socioeconomic factors such as migratory status and unemployment and to stress the differences among cultural minorities themselves. Classical literature on the interaction among cultural and social determinants of health identifies the above factors as influencing the health status of people of cultural minorities [20]. Works such as that of Douglas, Pacquiao and Purnell [33], among others, explore culture as a sense of belonging to a community, which some stakeholders viewed as a source of clashes with norms in healthcare and medicine.

Stakeholders profusely shared their views on the meaning of culture, especially the culture of minorities as opposed to theirs. In contrast, a clear definition of (one’s) culture was more evasive in MM users’ accounts. Participants in this group, when asked about their experience as part of a cultural minority, mentioned habits, external signs, and language, and they generally did not find any relation between such factors and their health status. More research is needed to elicit MM users’ reflections on how their cultural and social determinants relate to their health concerns [34] and their health status [35]. In our research, the majority of people with an MM background found culture to be in tension with equal treatment in healthcare. This is in agreement with Celik et al.’s [36] research on the Dutch context, whose results show how patients have a “neutral, disease-oriented approach”. Similarly, other studies in the US context conclude that cultural minorities’ perceptions of, and experiences with, healthcare provision do not include explicitly cultural competence as a key factor [37], but findings “predicted a desire for cultural matching” [24]. All works agree on the need for more research to better know how culture has an impact on the healthcare experience of both stakeholders and people with an MM background.

In our own research, stakeholders identified their own culture as a positive aspect for healthcare experience: liberal values such as laicism, scientific mindset, openness, universality, and equity are mentioned as components of healthcare, in line with what Global Health Europe [38] describes as European health values. When asked about minorities’ culture, most stakeholders identified factors such as religion, communitarian mindset, and conception of health as linked to illness and pain, as negatively influencing their acceptance of, and adherence to, a course of treatment or a particular care service. In this regard, Monteiro Mourão and da Costa Figueira Bernardes’ [39] literature review shows that “non-adherence among immigrants/ethnic minorities is mostly non-intentional, seeing as how it is associated with issues such as: low socio-economic conditions, language barriers and cultural mismatches”. Similarly, in our research, people with an MM background stressed difficulties accessing the system and language and communication as the most important when talking about cultural minorities and healthcare, while they also valued a multicultural social environment as positive to their healthcare experience.

Our participants discussed how what they identified as cultural differences could be a source of misunderstanding and conflict in their encounters with others in healthcare settings. The majority of stakeholders mentioned a climate of fear and distrust between them and people with an MM background, escalating to clashes and conflicts especially with some groups: Roma people, in relation to visiting hours, number of people in the hospital room, and behavior in general; and Muslim women, in relation to gender norms in clothing (the veil) and in treatment with male practitioners. Research shows the centrality of such groups in conflicts in healthcare, arguing that historical exclusionary processes locate Roma and Muslims as the ultimate Other [40]. Stakeholders’ responses about reasons for such misunderstanding and conflicts varied, from an interpretation of minorities’ culture as in tension with equal treatment for all, to a view of those conflicts as emanating from lack of mutual understanding.

On the other hand, most MM users did not identify culture as a source of conflict, and many stressed their own personal attitude as key in avoiding it. Generally, language difficulties were mentioned as important, especially by people living in the place of residence for less than six years, but they were not necessarily viewed as a source of conflict. However, problems with communication were so: for a majority of participants, not being listened to was a reason to get angry with, or to avoid, particular professionals. That communication played such a crucial role in all the interviews is in line with Messias et al.’s [41] work with formal and informal healthcare interpreters, which links the work of translation with communication and stresses implications in nursing such as the inclusion of “a shared commitment to language access and social justice”.

In line with this, when asked about discrimination and racist treatment in healthcare, people with an MM background did not recount any personal experiences as patients, but some of them identified language and communication problems, and problems with access while being undocumented, as in conflict with equal treatment and/or as discriminatory treatment. Instances of racist treatment were recalled, although with some doubts regarding naming them as such. More research is needed on the relationship of these doubts with implicit racial and ethnic bias in healthcare providers [42].

In contrast, some stakeholders clearly identified racist treatment in the form of racist comments both coming from professionals and patients, while others stressed universal access as key to avoid discrimination. However, some recognized that it is through misinformation and implicit prejudice that administrative and healthcare professionals discriminate against people with an MM background, especially against those in more vulnerable positions because they do not have documents and/or do not speak the language. Such exercises of othering resulting in people with an MM background receiving lower quality of care have been documented by Claeys et.al. [43] and Wanda et. al. [44], among others.

As we mentioned in the introduction, there is wide agreement that involving cultural minorities in healthcare processes is a key aspect for improving quality of care, but there is still a lot to do at a practical level [45]. When asked about the level of integration of people with MM backgrounds’ perspectives in the development, planning, and implementation of healthcare, none of the stakeholders recognized any, nor did they identify any involvement of MM people in the decision-making process, although some mentioned it at the user level. However, from the analysis of our interviews, we can discuss diverse notions of social diversity integration in care.

On the part of stakeholders, integrating social diversity in care generally meant adapting general services to cultural particularities. For the majority, the need for this adaptability was linked to the level of integration of the patient, especially to language proficiency. Two stakeholders went beyond cultural particularities and linked adaptability to the need for all patients to have an individualized care plan. In contrast, one stakeholder, while also valuing patient-centered care, perceived it as in tension with culture: for him, communitarian and/or religious norms clash with individualized care. This variety of views is in line with the findings of The Institute for Research in Superdiversity [22], which, among the gaps in the research on adaptation of health services to diversity, finds a lack of “detailed discussions of the ways in which potentially useful concepts such as patient-centering, quality of care or cultural safety play out in everyday health interactions”. All stakeholders identified systematic training of professionals in cultural diversity as key, but most of them insisted on the relationship of knowledge with attitude and personal experience with cultural diversity; some even made the first depend on the second to produce results.

In the case of people with an MM background, when asked about their integration in healthcare processes, they generally responded with doubts about the meaning of such integration. Participants with a long-established residence or born in the country of residence expressed doubts about the importance of cultural diversity’s integration in healthcare, because they understood it as in conflict with equal treatment in healthcare and with professionals’ actual disposition to care. However, as mentioned, participants in general stressed problems with language, communication, and access to the system, as well as lack of empathy, active listening on the part of professionals, and individualized care. This leads us to suggest that those are key factors of their experience with healthcare as part of cultural minorities, as some studies have shown [24,46,47].

Our participants described the aforementioned factors as barriers for cultural minorities in healthcare. Language was mentioned by all people with an MM background, and as noted above, some of them went beyond that and identified not being listened to, and not having the time to explain oneself, as key barriers. All the participants with less than six years of stay in their current place of residence (especially interviewees from Spain and Portugal) stressed the importance of barriers for undocumented people to have family doctors, in agreement with what authors such as Sahraoui [48] have documented across Europe. People with an MM background also mentioned as barriers problems shared by all populations, such as lack of information about how the system works, and waiting lists. One Polish patient, who is also a nurse in training, explicitly mentioned the small number of Polish healthcare providers in Belgium as a problem.

On the part of stakeholders, most of them identified increasing diversity within healthcare staff as a facilitator to social diversity integration. Given that, we can infer that they saw the lack of diversity as a barrier, although only one of them explicitly named it as such. This inference is in line with Saharaoui’s [49] work showing how, in spite of the differences among European countries, racialized workers are generally overrepresented in older-age care but underrepresented in clinical healthcare ones across Europe. Problems of trust and confidence due to language barriers, and especially communicational barriers, were key to stakeholders, as well as lack of time to dedicate to patients, difficulties in patients’ accessing primary care, and patients’ lack of general information about the system, results that are in line with other studies on barriers to, and strategies of, cultural competence in healthcare [19].

Together with diversity within staff, stakeholders identified other facilitators for the integration of social diversity in healthcare: translation teams and phone lines; good entry points to reach, communicate with, and listen to, people of cultural minorities; the use of cultural mediators, both experts and community’s leaders; and work on accessibility, healthcare collaboration with communities, and personalized and primary care. People with an MM background mentioned similar factors, while one person working with an NGO accompanying people in vulnerable situations added the need for training on how those people can exercise their right to healthcare. This vision of integration brings cultural minorities, and their exclusion from regular healthcare, to the fore of the struggle for universal and public healthcare [48].

### 4.1. Limitations

We acknowledge that excluding healthcare users with an MM background who could not communicate in the local language or in English may have limited the quality of our data. Specifically, it is likely that healthcare users with an MM background who are able to speak the local language or English (when English is not their mother tongue) are likely to be more acculturated and, therefore, have better access to healthcare services. In order to mitigate the impact of this inclusion criterion on our findings, we made an effort to include healthcare users with an MM background whose mother tongue was the local language, for example, Latin Americans in Spain.

We have reflected on the oversimplification embedded in the category “MM users”, as we know there is a great deal of diversity within it, due to place of origin and residence, migratory trajectory, social and economic conditions, as well as gender, age, and health condition, among many others. Our intention was not to homogenize culture, but to extract some common themes that are pertinent to the healthcare experiences of all cultural minority participants, such as the importance of communication and of accessibility, to name the most prominent.

Due to the situation derived from the COVID-19 pandemic, some of the interviews took place online. This may have hampered the communication between interviewer and interviewee, especially in the case of participants with an MM background whose mother tongue was not the local language. The researchers were aware of this and made a conscious effort to overcome the language barrier in these cases.

### 4.2. Strengths

The use of both groups to compare their vision and make an integrated analysis allows broadening and deepening the vision of stakeholders and users, providing a greater richness to the subject of study. In addition, this integrated analysis allows us to see the points of union between the highlighted topics with the objective not only to see the differences and attend individually each group, but also to see the positive and common points that both studied groups mention.

The use of the chosen methodology allows for a broader analysis of the issue in question, which is something that is emerging for the European community and that needs to be tackled as soon as possible: equity for all people in their access to healthcare systems and health.

## 5. Conclusions

In this paper, we have explored how key actors, such as stakeholders and people from an MM background, understand culture and describe their experience with healthcare. Results have shown that views of culture differ, especially on one crucial point: for stakeholders and MM users with long-established residence in the place of choice, culture is primarily what minorities have, and it has to do with family, community norms, religious beliefs, lifestyles, and habits. These components are perceived as in tension with healthcare norms and values, and they mediate in two key and related aspects of the relationship between MM users and healthcare providers: accessibility and communication. Those are precisely the two aspects that MM users identify as more important when talking about their experience: they are the main barriers they encounter to their integration in healthcare, and the arenas in which they feel misunderstanding and where conflicts arise. This leads us to conclude that integration of social cultural diversity in healthcare is a multifactor process that touches key aspects of healthcare for all populations, because it engages with discussions about patient-centered care, individualized care, active listening, proactive care, and universal access to care.

Another concluding remark has to do with the importance that the participants give to professionals’ training in cultural competence. MM users do not explicitly identify professionals’ cultural competence as key to their experience with healthcare, but they see professionals’ knowledge, personal attitude, and experience with cultural diversity as interrelated components facilitating their encounters with healthcare. It is important to mention that stakeholders agree with the importance of these aspects, which lead us to conclude that they are points of entry to work on integrating cultural diversity in healthcare.

## Figures and Tables

**Table 1:** Inclusion criteria for each group.

Stakeholders	MM Users
Individuals who represented and/or managed a patient association or an NGO dedicated to the promotion of social inclusion and cultural integration, or Individuals who represented and/or managed a healthcare professional association or council, or Individuals who represented and/or managed a local healthcare service including hospitals, trusts, specific areas, or zones, depending on each country, or Individuals who worked in the local or regional government, and who were responsible for the organization and management of healthcare, or Individuals with at least two-years of experience working in their respective positions.	Individuals with an MM background. Individuals who had lived in their current country of residence for at least 1 year. Individuals who were users of the national health service in each country. Adults aged 18 or over.
Individuals who agreed to the conditions of the study and gave informed consent to participate.	

**Table 2:** Themes and subthemes.

Themes	Subthemes
1. Concept of culture	1.1. Meaning of culture
	1.2. Impact of culture on health
2. Impact of culture on healthcare experience	2.1. Perception of own culture and its impact on healthcare experience
	2.2. Perception of the other’s culture and its impact on the healthcare experience
	2.3. Culture as a source of misunderstanding and conflict
3. Discrimination and racism	3.1. No personal experience
	3.2. Discrimination and equal access/treatment
	3.3. Racism treatment
4. Integrating Social Diversity in care	4.1. Importance of diversity in healthcare
	4.2. Education and training of healthcare professionals
5. Barriers accessing healthcare and limiting quality of care	5.1. Communication and language
	5.2. Institutional barriers
	5.3. Diversity within staff
	5.4. Resources
6. Facilitators	6.1. Communication and language
	6.2. Institutional orientation
	6.3. Diversity within staff
	6.4. Resources

**Table 3:** Sociodemographic and cultural characteristics of the participants.

		Stakeholders		MM Users	
		n	%	n	%
Gender	Female	9	64.29	13	68.42
	Male	5	35.71	6	31.58
Ethnic origin	White	11	91.67	5	26.32
	Black	1	8.33	6	31.58
	Asian	0	0.00	3	15.79
	Other *	0	0.00	5	26.32
Country	Spain	4	28.57	0	0.00
	Belgium	4	28.57	3	15.79
	Portugal	4	28.57	0	0.00
	Turkey	2	14.29	0	0.00
	Other **	0	0.00	16	84.21
Country of work	Spain	4	28.57	4	21.05
	Belgium	4	28.57	7	36.84
	Portugal	4	28.57	4	21.05
	Turkey	2	14.29	4	21.05
Religious Affiliation	Catholicism			7	36.84
	Islam			8	42.11
	Judaism			2	10.53
	None			2	10.53
Socioeconomic level	Low			3	15.79
	Middle			7	36.84
	High			9	47.37

* Other: Malinese 1 (5.26%), Mixed 2 (10.53%), Syrian 1 (5.26%) and Other 1 (5.26%); ** Other: Bangladesh 4 (21.05%), Croatia 1 (5.26%), Sudan 1 (5.26%), Indonesia 1 (5.26%), Poland 1 (5.26%), Saharawi Camps 1 (5.26%), Syria 1 (5.26%), Nicaragua 1 (5.26%), Guinea Bissau 1 (5.26%), Cape Verde 1 (5.26%), Cuba 1 (5.26%), Congo 1 (5.26%), Unknown 1 (5.26%).

## Data Availability

The data presented in this study are available on request from the corresponding author. The data are not publicly available because they are data of a sensitive nature containing the participants’ opinions. The European Commission’s support for the production of this publication does not constitute an endorsement of the contents, which reflect the views only of the authors, and the Commission cannot be held responsible for any use which may be made of the information contained therein.

## Supplementary Materials

The following are available online at https://www.mdpi.com/article/10.3390/ijerph181910503/s1, Table S1: COREQ guidelines.

## Appendix A. Topic Guide for Personal Interviews with Key Stakeholders and Decision Makers

Opening question We are interested in hearing about your perspective on transcultural healthcare. Can you tell me what you understand by transcultural care and what it means to you? Follow up questions - How do you think cultural difference affects health (i.e., ethnicity, nationality, religion, etc.)? What impact do you think being from a diverse cultural background has on patients/families/groups? - Are the perspectives of people from minority backgrounds reflected in health service development, planning and implementation? - Are people from diverse cultural backgrounds involved in the process of decision-making relating to the design and delivery of healthcare? - Are there specific services or pathways for patients belonging to cultural minority groups? If so, can you describe them? - Do you think the needs of patients belonging to minority groups are being met currently in healthcare services? - Do you think qualified nurses and other healthcare professionals are sufficiently prepared to deliver culturally appropriate care to patients from diverse cultural backgrounds? - What are the chief challenges or problems in the implementation of culturally safe healthcare currently? - What are the strengths or assets in the implementation of culturally safe healthcare currently?

## Appendix B. Topic Guide for Personal Interviews with People from an MM Background

Opening question I am interested in hearing about your experience as a health service user in this country. Can you describe which health services you have used in the past? Follow up questions - Could you identify any specific services or resources which have been offered or are available to you as belonging to a cultural minority? If not, have you missed any? - Do you think that opportunities for accessing healthcare services in this country may be diminished if you belong to a cultural minority? - Do you experience any barriers or difficulties communicating with the healthcare professionals? - Do you use any resources or strategies to overcome said communication barriers or difficulties? - Have your personal needs been addressed by the healthcare services? If partially, can you explain which needs are frequently met and which are frequently unmet? - Have you ever experienced discrimination or prejudice in the health service? - Do you think that the perspectives of people from minority backgrounds are currently being reflected in health service development, planning and implementation? - Do you think qualified nurses and other healthcare professionals are sufficiently prepared to deliver culturally appropriate care to patients from diverse cultural backgrounds? - Do you have any suggestions for improvement of the health services?

## Appendix C

**Table A1:** Summary of Themes and Subthemes and Example Quotes from the Personal Interviews with Stakeholders.

Theme	Subthemes	Selected Quotes
Concept of culture	Meaning of culture	“Life trajectory, place of origin, place where people have grown up (determines) beliefs and values” (S8) “Behaviors are based on cultural beliefs for centuries, so there are difficulties in changing them” (S14) “In the Western world, individual freedom is paramount, however, certain religions think more in “we terms.” They live more in communities and look at the community rather than themselves” (S2) “Because they depend very much on their culture, they defend their ideas, their respect for certain norms that they have deeply rooted and sometimes they clash, ok? with what has to be equal for all” (S5) “(There are some cultures, like Chinese, that) are very different, understanding is more difficult” (S7) “In my opinion, the strong point is the immense diversity that we have in our region, and country, we have different religions, different Ethnics, and different cultures” (S11)
	Impact of culture on health	“I think that people, as they have different ways of thinking, have different beliefs, different life habits and different types of eating. All these aspects have an impact on people’s lives and can affect these minorities negatively” (S12) “If you take the Roma ethnicity (…), their traditions and their lifestyle, diet has a lot to do with health, disease (…) There is a higher incidence of Epoc or asthma (…) People from Central Europe, the level of alcoholism (…) People of Latin origin, we also talk about traditions, habits in diet, that are harmful (for them) (…) (S6) “For a person of gypsy race (sic), one is sick when something hurts. They can have a 25/14 of blood pressure, 500 glycemia (…) but they do not feel sick. They associate pain to being sick. Culturally and educationally, they do not know about illness. This is a serious health problem” (S5) “Sub-Saharan people do not have the concept of chronic pathology, they live or they die” (S6) “Some people think that the disease process is in the hands of a God and not so much in the hands of the doctor. That causes to get therapeutic persistence more quickly” (S3)
Impact of culture in healthcare experience	Perception of own culture and its impact in healthcare experience	“We live in an open society and we are used to other cultures. There may not be enough attention to other cultures, but there is attention” (S2) “The adequation of concepts (of other cultures) to a secular healthcare, free of… that must be equal for all, is sometimes complicated” (S5) “There is always a strong autonomy of medical decisions (…) I don’t know if at the moment there are people from cultural minorities who are able to make decisions (about design and delivery of healthcare)” (S9) “We use the Biomedical Model a lot in the process of decision making. It is difficult to people from diverse cultural backgrounds to participate in their own decision making” (S10) “In certain cultures it is customary to surround patients a lot while we want to give our patients peace and quiet “ (S3) “A universal system, equal to all. Here you are going to have the same, the best assistance independently of your economic level” (S5)
	Perception of other’s culture an impact on healthcare experience	“Certain cultures are not used to sending family (members) to residential care centers (…) There are also forms of obesity and diabetes in different cultures because they do not get the appropriate medication for it (in time)” (S1) “People who object to how paramedics act towards them, namely people say that they don’t want a blood transfusion (…) (S9) “I wonder if people from other cultural backgrounds are also open to mental health services like people from Western culture. That may be a bias, I think they want to resolve that among themselves and are less likely to step up to mental healthcare. This is purely my own opinion and I cannot substantiate it either” (S4) “I know that they restore to healthcare in acute pathology situations and then follow-up in terms of primary healthcare does not happen (…) we lose track to these minority groups (S10)
	Culture as a source of misunderstanding and conflict	“What I notice that resonates with families is fear and distrust. They see things coming back that they are not used to so that feels fearful to them” (S1) “Sometimes there is nervousness or lack of patience. If something doesn’t go how they’re used to, there’s often a reaction like “At our place, it happens like this” (S2) “(Culture) has an impact especially when the person who belongs to a minority needs healthcare in a community where their cultural rules are not known, this may affect the behavior of caregivers who are, as always, considered normal, but for that community are sometimes even offensive” (S11) “(Conflicts) can be about simple things like respecting visitor hours. In certain cultures it is customary to surround patients a lot while we want to give our patients peace and quiet. It can also be about telling the truth, discussing subjects, diagnoses, deaths, euthanasia, taking medication, etc. (…) In the hospital it is not always easy for them to take other patients into account. When someone dies they dare to shout in the corridor, their experience is central at that moment” (S3) “In the case of gypsies, they want to be put in the ambulance and be immediately transported to the hospital, and paramedics have to do the initial screening and evaluation, and the gypsies do not realize that these acts are necessary, then conflicts and confusion arise” (S9) “It is been difficult for me (…) because you reach a level of vehemence on both parts, the lack of understanding, it is difficult for me to understand, for example, when there is someone sick and a lot of family members from gypsy race come, they are culturally like that but this clashes with norms and things that are not allowed for other people” (S5) “(A thing that is typical of us—Belgians—that other cultures clash on) is the relationship between man and woman. For example: if a man absolutely wants to go to the hospital with his wife for an examination and the staff asks the woman to wait in the waiting room, one has to accept it (…) It will definitely have an impact on the patient when (there is) too little time is taken to explain things (S2) “Muslim girls are quite resistant to be treated by a male doctor (…) particularly when they just arrived here, then they (adapt) (S8) “With Arab population, with Arab women, who come with their burkas (…) it irritates me, in the sense that unfortunately in my family I have seen my grandmothers and my mother fight for their autonomy and their dignity as women. And then to see something like that, it crashes me” (S5) “Sometimes they clash with your models or criteria, for example, in education, in mental health, in raising children (…) in our culture, there are things that are not acceptable and form them they are part of their cultural legacy (…) and they take it very badly when you say to them “ok, but in this culture we have laws, norms, and that is punishable, that is not allowed” (S7)
Discrimination and racism	No personal experience	“Healthcare providers will not turn away patients because of someone’s skin color. I feel that there is a positive evolution” (S2) “(We treat everyone), even the undocumented, and there is no problem” (S7) “I actually enjoy working with different cultures) (S1)
	Discrimination and equal access/treatment	“Undocumented people are more likely to get inferior care because they don’t understand each other well, for example. This is also the case with homeless people, they are more likely to be turned away because they cannot pay for the treatments, for example” (S2) “Boys have more problems, especially when they have not been long in Spain and they do not know the language, (staff) do not explain them things properly (…) they are not well assisted” (S8) “For example: Moroccan families take good care of each other, but that is not always the case. They need home nursing just as much (…) From the hospital perspective, you do notice that there are wrong prejudices. That’s also the case with residential and nursing homes, we automatically think that they have no need for that” (S3) “When a Gipsy family comes (…) there is a suspicion, a predisposition to conflict (…) “let’s see how they behave” (S5)
	Racist treatment	“I am amazed at how many racist comments are made, from both sides. I notice that that’s a strong presence with young people and within the healthcare system not everyone delves into cultures and there are often prejudices present that are passed down from home (…) During corona we also see that the “bad of man” comes out more and certain groups are targeted. This has always been the case in history” (S1) “The biggest challenge has to do with a smaller tolerance on the part of some people in Portuguese society, (…) Many Portuguese citizens have the perception that these people who come from outside, have more rights over their own nationals and, this is not quite clear, the Portuguese feel that they themselves are being discriminated against other people of minorities” (S9) “Sometimes there are problems when people share maternity rooms with people of color, of color, with people, you know… Sub-saharan Africans, there is suspicion on the part of the other person in the room. And the complaints are, look, it is very hard to even say it, complaints about uncleanness or odor, of course (it is) the smell of poverty” (S5)
Integrating social diversity in care	Importance of diversity in healthcare	“To provide healthcare to people with different ways of thinking, different religions” (S12) “Care should be generic, there should be an equal offer for everyone. Personal values and norms among different cultures must be taken into account” (S3) “The ability to adapt and try to meet what is the perspective of these cultural differences, not to alter, therefore, the good level of care” (S10) “Who belongs to a minority group? You can also consider older people a minority group, they often don’t know how or what to do. Then we are surprised that mistakes occur, but it is important to check off from time to time that everyone understands what is meant” (S1) “But what is background? In time, this is going to evolve. It’s about how long you are in a certain country and how you integrate into society (…) That has somewhere to do with background, but also with other skills. The place of residence also has an influence, what does a municipality do?” (S3) “(Their views come into play) when they are users, and they have needs. And as a sensible professional you realize them, or they reindicate them, if they are able to do it” (S7) “Feeling that their voice counts and that they can have input” (S4) “The easy thing is to adequate diet, or to have a multiconfessional space so people of other religions can pray” (S5) “It is only theory to say that we are going to care equally (…) I always say that the problem is the professional’ s adaptation to an individualized care plan (…) I am talking about Primary Care, those (individualized care and Primary Care) are two strengths (of the healthcare system)” (S6) “There is no such thing as a patient-centered patient anymore, the family is part of it, and religion is also central. Then you wonder if you are giving good individual care. If a patient says “I don’t want pain, give me morphine,” and the family and religion stand above that, then the patient’s wishes are suppressed” (S3)
	Education and training of healthcare professionals	“Yes, it’s important to get an education. If you see a mom coming in with 4 kids, you can get nervous on the one hand and deal with that situation in a calm way and point out to them that next time it’s best for her to come alone. Experience helps in such situations” (S2) “First of all, the need in this area should be recognized. Then, after the human resources and training process with necessary financing and infrastructure, the employment of personnel trained in this filed within the existing health system is required” (S13) “(There is training) individually, but going beyond voluntarism, I think there is not. There is no field, no workshop, no training, no signatory course that teaches you this (…) I think there is an idea, offering a continuous training program” (S7) “Knowledge has a lot to do with our attitude. If you are not educated or have no knowledge about different cultures, your attitude also suffers. If you are used to different cultural differences, you are automatically going to have a more open attitude” (S2) “Look, I think that we have training resources, (what we need is) changes in attitude in professionals to better assist (people), we should be more flexible” (S6) “There is not so much training as quality of the person” (S8)
Barriers	Communication and language	“Mutually, there are problems based on trust (…) there are difficulties in providing services due to communication and trust problems” (S14) “Yes, language is an important means of communication. We often work with interpreters, but then you often have the aspect of trust again. They are also strangers to patients, so if you don’t know the language, you also have to trust them” (S1)
	Institutional barriers	“In the care sector, time is the problem. If you don’t make time for people who are not used to care in Belgium, combined with the language barrier, this can cause problems” (S2) “I find it striking that certain people do not have a regular family doctor. The PCSW (public welfare) could possibly help with that by looking for GPs who still have available places, so that every family has a GP available and they always have access to care” (S4) “Digitalization causes many people to disappear from the radar (…) I think the healthcare system is not reflective of the population. There are only a limited number of educated people who can take care of themselves” (S3) “I think there is no very big involvement in decision-making, migrants are little involved I would say almost zero” (S11)
	Diversity within staff	“You notice that also in other institutions such as education, there are also few people with a different background working there” (S4)
	Resources	“That’s very much related to the poverty issue. There should be initial efforts to get people out of poverty like making sure they have a good job and place to live” (S3) “If they don’t get the education around it (health and healthcare) as much, you notice bigger problems there” (S1)
Facilitators	Communication and language	“Of course we can’t have a translator in every hospital in every health center but maybe we should bet a little more on these translation support telephone lines in different languages in a 24-h…” (S10) “Communication and education. Especially being able to reach people in an appropriate way. The right people need to be addressed and find good entry points” (S1) “In these communities the figure of the mediator should always emerge to be a facilitator in communication between health teams and minorities, in order to make better integration by the one to communicate better. This mediator may play a very important role in the exchange of information and, if health brings benefits to the provision of care users of minorities, it could establish points the patient, physicians and nurses for a better understanding of the situation” (S12)
	Institutional orientation	“The cooperation and integration of the different cultures. We have to keep believing in it and we can learn a lot from each other (…) If further research is done on that, the result would be wonderful” (S1) “Yes, a growth in empathy. You have to be able to imagine very well why someone wants or doesn’t want certain things (…) the care does not immediately become better, but existentially it does” (S3) “Accessibility and personal assistance, talking about Primary Care, these are the strengths” (S5)
	Diversity within staff	“Diversity within social workers. More people from cultural backgrounds need to be hired. Patients look up to them and have confidence in them. Language skills are also a challenge” (S3) “Yes, in NGOs and associations there are many people with diverse origins, which has an influence on the policy and the way of approach. The habit of working with people of a different culture also plays a role” (S2) “I think that what we have left if 20 years and another generation (…) there are no problems (when people) are integrated in this kind of work” (S5)
	Resources	“It is important to have a point of contact for people from different cultural backgrounds. This point of contact can serve as a navigation that can help the patient further. For the vulnerable groups there should be proactive care, people who are actively involved in the care of certain patients and who follow them up systematically” (S3) “A de-digitization program. Not everything has to be digitized, but they have to find a way that vulnerable people can still access care. I also believe for multiculturalism with the new primary care zones (system that has been implemented to organize care), there will be an integrated reception” (S3) “In recent times I have noticed that there are already some steps to be taken, particularly when in the crisis of the Covid19 pandemic, it was careful to pass the information on not only in the Portugese language but also in languages that are already common in our country, namely Mandarin, Spanish, English, or French” (S9) “Instead of translation, you need an intercultural mediator. Because is not only the words that he says, it is the meaning in his contexts” (S5) “There is a risk of being burdened as a workload in addition to existing tasks” (S13)

## Appendix D

**Table A2:** Summary of Themes and Subthemes and Example Quotes from the Personal Interviews with MM Users.

Theme	Subthemes	Selected Quotes
Concept of culture	Meaning of culture	“Often they come to me and ask “Is this true?” and then I give them more information about the topic (…) People ask me why I still wear it (a yarmulke) (…)” (M5) “Each doctor asks me “where are you from”? (M10) “Some Jewish people prefer to not go to the hospital on Saturday and Friday in the afternoon (…) There are also people who don’t want to have contact or shake hands with the opposite sex (…) these habits are typical of our culture” (M7) “I wear the veil, but they do not look at me as if I was alien, I do not know (M11) “We were raised Dutch-speaking. We came here and then my father told us to adapt and speak Dutch” (M5) “I try to talk to my African friends always in Portuguese, in order to better train the Portugese language and so we make amends to each other” (M13) “What I see is that, in the healthcare sector, they see all people as equal, there is no minority, or a black or a white person” (M11)
	Impact of culture on health	“Most people are not aware that specific diseases are more common in certain populations (…) For instance: breast cancer, the PSCA gene is more common in the Jewish population. I don’t think it is well-known. If it was more known, people would get screened more” (M7) “If you are a non-native speaker it can be different. Often it’s about misunderstanding” (M1) “For the migrant people that are here (Spain) for less than a year, there are a lot of difficulties (…) First, to regularize its situation, second, to think of oneself as having rights. There could be a part that has to do with the place of origin, where maybe the conception of rights’ bearer is not internalized as much” (M8)
Impact of culture on healthcare experience	Perception of own culture and its impact on healthcare	“It is difficult (for healthcare staff) to be aware of small customs of other cultures (…) I do not think they should be prepared for such small things (…) Perhaps provide activities for patients who receive few visitors, definitely during the corona pandemic. The food could perhaps also be improved” (M4) “I am not religious (…) I have a more objective view (…) Life is precious and if you can save someone you are allowed to break all the (religious) rules” (M7) “People think that if they restore to public healthcare everything is going to be perfect, they are going to get assistance even with the smallest details, and it is not like that, so people lose confidence” (M9) “I always try to speak in Portuguese, to try to improve my communication. I avoid speaking Creole with my African friends’’ (M12) “Everyone who has a family doctor have their needs met, the problem is that there are many Africans without a family doctor” (M15)
	Perception of others’ culture and its impact on healthcare experience	“Brussels is even more multicultural so I expect the problems about culture would be even smaller” (M7) “Istanbul is more cosmopolitan so the health staff have more exposure to the multicultural community. However, central Anatolia is not as cosmopolitan. The health professionals need language proficiency and cultural awareness in order to deal with foreign patients. They may not be adequately sensitive at times” (M18) “There is a mantra that the immigrants use healthcare more than anyone else (…) and that generates that immigrants do not think of themselves as worthy of such assistance” (M8)
	Culture as a source of misunderstanding and conflict	“No, I have never experienced a problem in a hospital or even in school. A lot of people have had questions about my culture, but I have not experienced any problems” (M4) “I am in counseling with Vaga (local Mental Health Center) and there I do feel that we are not on the same wavelengths. However, that has nothing to do with (cultural) background” (M1) “Apart from my name, I don’t think many caregivers know that I have a Jewish background. I don’t experience any difference. The ones who are aware I am Jewish or see my name, do not respond differently and if they do, they ask questions about my religion so it is only in a positive way. I have never been in a negative situation about my culture” (M7) “Wherever I went, I’ve always been appreciated no matter who I am and where I’m from. That’s how I approach other people too. That also has something to do with it. I think the way you approach others. I may have a dark skin color, but I’m not crazy. laughs]” (M5) “I can imagine a Moroccan or African boy having problems with that, but I don’t experience those problems”. (M5) “My language is not perfect after a few years, but if people notice that you are making an effort, they are friendly”. (M6) “It is about what the patients expect (…) you can’t expect to be treated as a Muslim if you live in a Christian country (…) Faith and care are separate, I think. I don’t believe in discrimination” (M5) “To listen and be listened to. If you are going to talk to a doctor, and he or she is in another world, the patient gets angry, because you see in the doctor’s face that he/she is not with you” (M11)
Discrimination and racism	No personal experience as patient	“(I have not experienced discrimination), not from the staff. Sometimes there are other patients who are discriminated and/or prejudiced” (M1) “I know friends that do internships and I have heard that some companies reject them because of their culture” (M4) “(I faced) discrimination as an intern, but as patient no (…) Everyone is equal and everyone gets the same access to health services. ” (M6) “I can’t speak on that. I also used to be the only child with a different skin color, but never missed out on help. I hope not, but I dare not rule it out. If that were the case, I would be shocked. It is possible that I am discriminated against because of my name, but once I explain it I don’t experience any problems. I often have to explain myself where I come from, but I’m used to that from childhood. At school they used to ask where I and my parents come from. My daughter also has to explain it in turn” (M5)
	Discrimination and equal access/treatment	“We have doubts if they could assist us (…) we are not Spanish (…) at the counter they said that we must pay for private insurance because I do not have documents (…) I do not think it is an obligation, but I feel it is a right to belong to public healthcare” (M10) “If we do not have a family doctor or used card it is very difficult to buy medicines without a discount. I think foreigners should have this possibility” (M12) “(With language) some people (staff) don’t have much patience in that (…) (A foreign name can cause to offer help) less enthusiastically” (M2) “(Staff) think that because you are in Spanish soil you have to know the language (…) (There is misinformation) and people are not stupid, they see their rights violated, things are denied, and this could be discriminatory (M8)
	Racist treatment	“I do not know if this racism or not, but because she was a Muslim, a girl wearing the veil (…) that person (the doctor) wants to end it, does not respond to questions, maltreat, and such” (M9) “It is a more personal thing, I think. People would like to work with Spaniards better than Africans” (M9) “I did volunteer work at the Sint-Lodewijk rest home in Schilde. The patients appreciated the help, but they were racist. That is also due to the generation (…) I had to feed in the retirement home one time and there was couscous on the menu. There was a lady at the time who was not very open to this. “That’s from the macaques”, she said.” (M2)
Integrating social diversity in care	Importance of diversity in healthcare	(…) Do those minorities need different care than Belgians? I wonder. I don’t believe that a nurse looks at your origin, they want to make someone better. At that point, there is no room for pigeonholing” (M5) “I am not sure. I don’t think it would have that much of an influence. In all cultures there are people on both sides. You will find Jewish, Muslim, Hindu Doctors, etc. I don’t think that should be an argument. If you really need help, you will always be able to find someone. You should not judge access based on religion, when you need help you should go to the first person that can help you” (M7) “It would be better if help were provided more quickly. Using a motorcycle instead of a car can sometimes be quicker” (M4) “There is no tact, there is no… I see the healthcare professional as any other civil servant, and he lacks the ability to think that we are not identical, to think that you have a person in front of you who does not have to adapt herself to you, not you to her, but you must find common ground (…) (M8) “On the one hand, I think it’s good that Polish doctors are available in Belgium, but on the other hand, a few Polish doctors are not enough to help the whole Polish community. People of my age are able to learn a new language faster and will also go to Belgian doctors, but when you are older it is not so easy and you prefer Polish doctors” (M6) “If for example I go for a mammogram and they ask me (…) and I do not understand the question, they explain me perfectly, they pose a simpler question so I can answer it. Or I give the answer they are looking for (…) “ (M7) “One small point of work is that the caregivers are not always empathetic. Of course, it is very busy now so I understand that there is often little time, but there is still room for improvement in terms of individual treatment so that the patient feels understood and worthy. For example, they should not be afraid to ask questions. Other than that, I have no major minuses, I only have pluses (laughs) (M6) “Yes, I think it is something that is lacking in our current healthcare. The services are not personalised and remain very general, despite the fact we live in a multicultural society” (M7) “Everyone should have mandatory insurance so that everyone is equal and entitled to the same care. It should not be allowed for someone who has more money to be better cared for. We all have a right to be taken care of” (M5) “I actually think it’s positive when many different cultures work together. The diversity ensures that people are helped better. People of different origins working as nurses or bakers, for example (…) that would improve care” (M2) “When I go to a hospital, it is not necessary to have a Jewish caregiver who helps me. The staff is often multicultural so it gives me the feeling that everyone is welcome” (M4) “I think most hospitals have a kosher room. On Saturdays, we are not allowed to switch the lights on. Most hospitals are aware of this and do what they can” (M4) “First of all, I know that the practitioners switch to the language of the patient’s preference so they usually speak French with Jewish patients. They easily adapt to the language. Some Jewish people prefer to not go to the hospital on Saturday and Friday in the afternoon. Usually the doctors are quite flexible in adapting to our culture” (M7)
	Education and training of healthcare professionals	“I am very satisfied with that (healthcare professionals trained to provide care to people who have different cultural backgrounds)” (M2) “I do not know if that is important. They already have a lot to study” (M4) “Yes (there is room to improve healthcare in Belgium), definitely in terms of communication. Soon we will have an online session on international communication skills, which will also be given in English. It’s about the way we communicate with the patient” (M6) “There is no course on culture, or trans-cultural (…) so it is the sensibility you have as an individual, if you wanted to get a training yourself, or your personal experience (…) I think there should be internal training for professionals” (M8) “There are a lot of people that have empathy. They put themselves in others’ shoes (…) We can say that 85% have that empathy, they understand that, to cure a sick person (they need to) give her love, more than give her medication” (M11) “I don’t think it is adequate. In the public healthcare services, qualified nurses as well as other healthcare professionals are available but they do not make any special effort to reach you because you are a foreigner. They still need to train themselves to provide the efficient medical services to the people belonging to diverse cultural background” (M16)
Barriers	Communication and language	“I have faced difficulties in communicating with the healthcare professionals. Here, Turkish language is the official language and professionals didn’t had enough skills in English especially in the public hospitals” (M16) “People who don’t work here (in hospitals) have a harder time with the terminology. For example, I speak English, but I also have to look up the meaning of words regularly. That’s why we sometimes need interpreters. That is a small disadvantage, but on the other hand you can’t provide an interpreter for all nationalities.” (M7) “They do not allow you to explain yourself enough (…) I drop off from that doctor” (M9)
	Institutional barriers	“(…)when you come here (Spain), this happens a lot, every person with a uniform, be it police be it healthcare uniform, exercises a power, a reaction (on the part of the migrant) of submission and fear (…) there are civil servants in one rail, and in another the migrant population, and there are clashes” (M8) “In the beginning, when I went to register myself in the primary care center they told me why I did not pay for private insurance if I had no documents” (M10) “It’s just hard to have a family doctor” (M12)
	Diversity within staff	“Often my language is not good enough to communicate with Belgian healthcare providers, so sometimes I go to Polish healthcare providers, but because there are only a few Polish healthcare providers, they are often very busy so the quality of care is not so good. That’s the reason why we changed GPs”. (M6)
	Resources	“Sometimes there is a lack of information regarding appointments from national electronic appointment system for public hospitals” (M19) “I experience long waiting lists on a regular basis (…) I had a difficult time (…) many people experience that” “I had a bladder infection last year (…) I had explained my situation on the phone to the Polish GP and went for a consultation afterwards. I missed the contact and interaction with the doctor which made me feel like one of many patients” (M6)
Facilitators	Communication and language	“There are more people from migrant origin who are doctors, nurses, there is a change that is going to happen (…) But we are talking about a very long process, one that is very slow. What can be done in the meantime? Think about what we are doing now and change it, for example, with translators (…) cultural mediators, social educators” (M8)
	Institutional orientation	“Training on how you can exercise your right as a user of a public service. Companionship, intercultural mediation, but those are strategies that imply money, an effort to try to integrate those people in the good sense of the word, to integrate them in their rights” (M8) “There is a need for more individual support (…) individualized care” (M2)
	Diversity within staff	“I actually think it’s positive when many different cultures work together. The diversity ensures that people are helped better. People from different origins working as nurses or bakers, for example” (M2) “Probably more health staff should be engaged and multilingual ones would be an added asset” (M19)
	Resources	“People reach for their cell phones very quickly. People who are older, like my parents, rely on their children to help them (…) the Internet is also used, often the Facebook group is used and people help each other (with documents) Real resources are not used much, but we mainly help each other” (M6) “Accompaniment, I go with a companion, or with my husband, or I call another person to intermediate between us (her and the doctor) (…) I say words in English, in French, I draw, I make an effort until I reach my objective” (M11) “It is interesting that the Jewish community has its own ambulance service, they work together with hospitals. This increases the access and collaborations” (M7)
