# Supplementary material for: Perception and Experience of Transcultural Care of Stakeholders and Health Service Users with a Migrant Background: A Qualitative Study
Source: Int J Environ Res Public Health. 2021 Oct 7;18(19):10503. doi: 10.3390/ijerph181910503 (PMC8508346; doi:10.3390/ijerph181910503)
Supplement: Supplementary file 1 [file ijerph-18-10503-s001.zip › ijerph-1387977-supplementary.pdf]

Supplementary Table S1. COREQ guidelines

№	Item	Guide questions/description	Page number
**Domain 1: Research team and reflexivity**			
Personal Characteristics			
1.	Interviewer	Which author/s conducted the interview or focus group?	Title page
2.	Credentials	What were the researcher's credentials?	Title page
3.	Occupation	What was their occupation at the time of the study?	Title page
4.	Gender	Was the researcher male or female?	Title page
5.	Experience and training	What experience or training did the researcher have?	Title page
Relationship with participants			
6.	Relationship established	Was a relationship established prior to study commencement?	p.4
7.	Participant knowledge of the interviewer	What did the participants know about the researcher?	pp.4, 5
8.	Interviewer characteristics	What characteristics were reported about the interviewer/facilitator?	p.5
**Domain 2: study design**			
Theoretical framework			
9.	Methodological orientation and theory	What methodological orientation was stated to underpin the study?	p.3
Participant selection			
10.	Sampling	How were participants selected?	p.4
11.	Method of approach	How were participants approached?	p.4
12.	Sample size	How many participants were in the study?	p.4
13.	Non-participation	How many people refused to participate or dropped out? Reasons?	N/A

Setting			
14.	Setting of data collection	Where was the data collected?	p.5
15.	Presence of non-participants	Was anyone else present besides the participants and researchers?	p.5
16.	Description of sample	What are the important characteristics of the sample?	pp.5, 9-10
Data collection			
17.	Interview guide	Were questions, prompts, guides provided by the authors? Was it pilot tested?	pp.6-7
18.	Repeat interviews	Were repeat interviews carried out? If yes, how many?	N/A
19.	Audio/visual recording	Did the research use audio or visual recording to collect the data?	p.5
20.	Field notes	Were field notes made during and/or after the interview or focus group?	N/A
21.	Duration	What was the duration of the interviews or focus group?	p.5
22.	Data saturation	Was data saturation discussed?	N/A
23.	Transcripts returned	Were transcripts returned to participants for comment and/or correction?	N/A
**Domain 3: analysis and findings**			
Data analysis			
24.	Number of data coders	How many data coders coded the data?	p.7
25.	Description of the coding tree	Did authors provide a description of the coding tree?	p.8
26.	Derivation of themes	Were themes identified in advance or derived from the data?	P.7
27.	Software	What software, if applicable, was used to manage the data?	N/A

28.	Participant checking	Did participants provide feedback on the findings?	N/A
Reporting			
29.	Quotations presented	Were participant quotations presented to illustrate the themes / findings? Was each quotation identified?	Results
30.	Data and findings consistent	Was there consistency between the data presented and the findings?	Results
31.	Clarity of major themes	Were major themes clearly presented in the findings?	Results
32.	Clarity of minor themes	Is there a description of diverse cases or discussion of minor themes?	Results
